# Measuring Effort–Reward Imbalance in School Settings: A Novel Approach and Its Association With Self-Rated Health

**DOI:** 10.2188/jea.JE20090057

**Published:** 2010-03-05

**Authors:** Jian Li, Li Shang, Tao Wang, Johannes Siegrist

**Affiliations:** 1 Department of Medical Sociology, University of Duesseldorf, Duesseldorf, Germany; 2 Department of Safety Engineering, University of Wuppertal, Wuppertal, Germany; 3 School of Public Health, Kunming Medical University, Kunming, China; 4 Yunnan Provincial Department of Education, Kunming, China

**Keywords:** effort–reward imbalance, psychosocial school stress, self-rated health, adolescents

## Abstract

**Background:**

We attempted to apply the model of effort–reward imbalance (ERI) to school settings in order to measure students’ psychosocial stress and analyze its association with self-rated health in adolescents.

**Methods:**

A cross-sectional survey was conducted in Kunming, China among 1004 Chinese students (468 boys and 536 girls) in grades 7 through 12, using a 19-item effort–reward imbalance questionnaire.

**Results:**

Satisfactory internal consistencies for the scales for effort and reward were obtained; the value for the scale for overcommitment was acceptable. Factor analysis replicated the theoretical structure of the ERI construct in this sample of Chinese students. All 3 scales were associated with an elevated odds ratio for diminished self-rated health, and the effect was strongest for the effort–reward ratio, as predicted by the theory. Sex and grade differences were also observed.

**Conclusions:**

The ERI questionnaire is a valid instrument for identifying sources of stressful experience, in terms of effort–reward imbalance, among adolescents in school settings.

## INTRODUCTION

Schools are a powerful psychosocial environment for most children and adolescents worldwide. Despite changes in the types of schools, locations, and classes during students’ educational trajectories, students experience a rather consistent basic structure of relationships between the requirements of study and their capabilities, resources, needs, and coping strategies. These relationships are embedded in group dynamics within classes and within specific peer groups. Several studies have documented unfavorable effects on performance and well-being among students who were exposed to adverse material and psychosocial school settings in both Western^1^^–^^6^ and Asian countries.^7^^–^^14^ However, because the notion of an adverse psychosocial school environment has rarely been defined in theoretical terms, it is difficult to compare and generalize findings from such studies and to test explanatory hypotheses. There are some obvious similarities between school and work settings. Both represent formalized organizations with defined social norms and roles, and the continued meeting of demands is expected in both organizations. Moreover, demands, control, and reward are experienced in non-symmetric relationships, with salaried employees (students) having less power than employers (teachers).^15^^–^^17^ Thus, despite obvious differences (see below), it is tempting to explore the extent to which theoretical models that were developed in the context of work can be applied and adapted to the school environment.

Internationally, two such models received particular attention in the recent past with regard to health and well-being: the demand–control model,^18^ and the effort–reward imbalance model.^19^ The former model posits that highly demanding work, in combination with low decision latitude and a low level of a control over performance, adversely affects health and impairs learning and personal development.^20^ The latter model focuses instead on fairness of exchange in terms of the balance between efforts spent and subsequent rewards received. If high costs spent are not reciprocated by adequate gains, negative emotions are elicited, with adverse long-term consequences for health and well-being.^21^ The former model has been applied to school settings in a Swedish study^2^; however, to our knowledge, no such attempt has been made with regard to the latter model.

It is important to address the differences between school and work settings from a social epidemiological perspective, in particular with respect to the theoretical model of effort–reward imbalance. In the workplace, efforts spent are part of the work contract, whereas efforts at school represent reactions to less explicit expectations from the school and from parents. Traditionally, Chinese parents have very high expectations, requiring their child to behave like a “dragon” or “phoenix”.^14^ Secondly, rewards at work concern money, esteem, promotions, and job security, whereas rewards at school are related to good academic performance (direct feedback), esteem (from both teachers and schoolmates), and increased prospects due to education (a promising future through education). The third component of the model, overcommitment, although of interest, may be less important in adolescents than in adult working populations, because the former have a less established pattern of coping with recurrent demands (overcommitment).^15^^–^^17^

We conducted a psychometric test of a newly developed standardized self-administered questionnaire that assessed student experiences of effort–reward imbalance in school settings. In addition, we explored associations with self-rated health. Providing a theory-based measure of an adverse psychosocial school environment should prove useful in attempts to develop and implement “healthy schools,” as proposed in the WHO’s international Health Behaviour in School-aged Children (HBSC) program.^16^^,^^17^

## METHODS

### Study design and sample

A cross-sectional study was conducted in 4 general schools located in different socioeconomic areas of Kunming, a large city in southwestern China. The study protocol was approved by the Ethical Committee of the Kunming Medical University, and the research was performed in accordance with the Declaration of Helsinki. A total of 1249 students from grades 7 through 12 were invited to participate. The questionnaire was completed by 1089 students (response rate, 87.19%) recruited from a total of 21 classes. Three of the 4 schools provided instruction to students in grades 7 through 12, but 1 school taught students in grades 10 through 12 only. According to the International Standard Classification of Education (ISCED) by the United Nations Educational, Scientific and Cultural Organization (UNESCO),^22^ grades 7 through 9 belong to Level 2—lower secondary education or the second stage of basic education (commonly called middle school)—which is still compulsory in China; grades 10 through 12 belong to Level 3—upper secondary education (commonly called high school)—which is no longer compulsory in China.

For this study, 1 class was randomly chosen from every grade in every school included. Out of the 1089 respondents from the 21 classes, a total of 1004 questionnaires without missing values remained for data analysis. Thus, this report is based on a sample of 468 boys and 536 girls with a mean age of 15.9 years. There is no evidence that this sample was biased in terms of selection criteria. The 4 schools were selected from different socioeconomic areas within the city in order to balance socioeconomic inequalities. Nevertheless, the proportion of students completing advanced grade levels is much higher in middle-class and upper-class population groups in China (see Table 1).

**Table 1.** Characteristics of the study subjects (*n* = 1004)

Variables		Boys (*n* = 468)	Girls (*n* = 536)
		*n* (%)	*n* (%)
Age (mean ± SD)		15.84 ± 1.91	15.87 ± 1.77
Daily hours of homework, ​ Monday to Friday (mean ± SD)		2.56 ± 1.14	2.72 ± 1.11
Daily hours of homework, ​ weekend (mean ± SD)		3.31 ± 1.55	3.54 ± 1.53
Daily hours of TV watching, ​ Monday to Friday (mean ± SD)		1.18 ± 1.13	1.06 ± 1.04
Daily hours of TV watching, ​ weekend (mean ± SD)		2.61 ± 1.69	2.67 ± 1.64
Grade level	7	73 (15.60)	53 (9.89)
	8	85 (18.16)	95 (17.72)
	9	76 (16.24)	98 (18.28)
	10	75 (16.03)	103 (19.22)
	11	65 (13.89)	75 (13.99)
	12	94 (20.08)	112 (20.90)
Smoking	No	407 (86.97)	530 (98.88)
	Yes	61 (13.03)	6 (1.12)
Alcohol drinking	No	333 (71.15)	480 (89.55)
	Yes	135 (28.85)	56 (10.45)
Physical inactivity	Active	165 (35.26)	125 (23.32)
	Inactive	303 (64.74)	411 (76.68)
Family wealth	High	208 (44.45)	207 (38.62)
	Average	219 (46.79)	299 (55.78)
	Low	41 (8.76)	30 (5.60)
Self-rated health	Good	318 (67.95)	312 (58.21)
	Fair	123 (26.28)	198 (36.94)
	Poor	27 (5.77)	26 (4.85)

### Measures

A new 19-item questionnaire measuring the model’s 2 extrinsic components—effort (5 items including schoolwork and expectations) and reward (11 items including academic performance, esteem, and educational prospects)—and the intrinsic component overcommitment (3 items) was used for the study (some items were presented in reversed form, and are marked with an * in the Appendix). Using the study protocol of the international WHO-HBSC study,^23^ some items were extracted from the optional package for school settings (marked with an † in the Appendix) and added to the ERI questionnaire. Most of the items (12 out of 19) on the questionnaire mirrored the original wording of the established scales,^21^^,^^24^ but significant differences between the school and work environments had to be taken into account. Respondents marked items using 5-point Likert scale ratings from strongly disagree to strongly agree.

In addition to sociodemographic data (sex, age, and family wealth), selected study-related time consumption and health-adverse behaviors were monitored, according to the study protocol of the international WHO-HBSC study,^23^ in particular the hours for homework and TV watching, and the prevalence of cigarette smoking, alcohol drinking, and physical inactivity. Moreover, current self-rated health was assessed by a single item, “How would you say your health is?”, using 4-point Likert scale ratings of excellent, good, fair, and poor. It has been reported that self-rated health is a relatively stable construct during adolescence, and is significantly prospectively associated with general well-being, disability, healthcare attendance, and health-compromising lifestyle factors.^25^

### Statistical analyses

Following descriptive analyses, we used established procedures to test the psychometric properties of the scales measuring the adapted effort–reward imbalance model. Firstly, scale means were computed, and *P* for trend with analysis of covariance was used to examine sex and grade level differences. Secondly, item-total correlations and Cronbach’s alpha (including scale alpha and alpha if item is deleted) were calculated to assess the internal consistency of the scales. Thirdly, the factorial structure of the 19-item questionnaire was explored using principal axis extraction and varimax rotation. In addition to exploratory factor analysis, confirmatory factor analysis was performed to test the dimensional structure of the theoretical model, and goodness-of-fit indices were calculated for the scales.

Finally, we conducted multivariate ordinal logistic regression analyses using self-rated health as a criterion variable to test associations of effort–reward imbalance at school with reduced well-being. It was recently reported that ordinal logistic regression has more explanatory power than ordinary binomial logistic regression.^26^ Therefore, self-rated health was combined into 3 ordinal categories: good health, fair health, and poor health. In addition to testing single scales, tertiles of the ratio between the 2 scales, ie, effort (nominator) and reward (denominator, adjusted for an unequal number of items), were entered into the analysis to represent quantitatively the core theoretical notion of imbalance. The same procedure was done with the logarithmic ratio, where the effects of 1 standard deviation were estimated.^27^ Several ordinal logistic regression models were calculated adjusting incrementally for age, sex, grade level, health-adverse behaviors, and family wealth. Also, sex-specific and grade-specific analyses were performed. We verified the model fit by using the Hosmer–Lemeshow goodness-of-fit test and the score test of the proportional odds assumption. In all cases, the models were appropriate for the data with good fit (*P* > 0.05). All calculations were done with the statistical program SAS 9.2 (SAS Institute, Inc, Cary, NC).

## RESULTS

Table 1 shows the main characteristics of the study population by sex. There were no substantial differences in age or time spent watching TV between boys and girls, but girls spent more time on homework. The prevalences of smoking and alcohol drinking were much higher among boys than among girls; however, more girls than boys were physically inactive. There were slight differences with regard to family wealth and self-rated health: fewer boys than girls were from families with average wealth, and more girls than boys rated their health as fair.

The scale means and standard deviations are presented in Table 2. Girls had a higher score for overcommitment than did boys; there were no sex differences in effort or reward. Notably, significant differences were revealed by grade-specific analyses. As compared to middle school students (grades 7–9), high school students (grades 10–12) reported much greater effort and much lower reward and overcommitment.

**Table 2.** Means and standard deviations of the scales of the 19-item Effort–Reward Imbalance questionnaire, by sex and grade level

		Effort	Reward	Effort–reward ratio	Overcommitment
Sex	Boys	17.40 ± 3.48	39.36 ± 5.92	1.01 ± 0.31	9.21 ± 2.43
	Girls	17.72 ± 3.27	39.32 ± 5.48	1.02 ± 0.28	9.63 ± 2.39
*P* for trend		0.42	0.58	0.94	<0.01
Grade	7	15.44 ± 4.18	42.36 ± 6.60	0.85 ± 0.35	9.56 ± 2.47
	8	16.47 ± 3.10	41.56 ± 5.86	0.90 ± 0.25	9.61 ± 2.51
	9	18.13 ± 3.10	41.43 ± 5.09	0.98 ± 0.23	10.09 ± 2.44
	10	18.00 ± 3.36	37.20 ± 5.02	1.10 ± 0.34	9.21 ± 2.44
	11	18.44 ± 2.84	36.40 ± 4.80	1.14 ± 0.26	9.04 ± 2.23
	12	18.39 ± 2.77	37.63 ± 3.99	1.09 ± 0.22	9.12 ± 2.27
*P* for trend		<0.001	<0.001	<0.001	<0.001

In Table 3, item-total correlations for each scale, together with scale alpha and alpha if item is deleted, are displayed as measures of internal consistency. All item-total correlation coefficients in our results were larger than 0.30, which is considered satisfactory. With the exception of the scale overcommitment (3 items only), Cronbach’s alpha values were satisfactory. Table 4 shows the factorial structure of the questionnaire, using exploratory factor analysis. The 4-factorial solution clearly fits the hypothesized structure of the ERI questionnaire. The second factor clearly indicated effort, and the fourth factor was associated with overcommitment; reward items were loaded on the first factor (referring to esteem and educational prospects) and the third factor (indicating academic performance). The amount of variance explained by the 4 factors was moderate (36.4 percent). Furthermore, the goodness-of-fit indices (GFI) in the confirmatory factor analysis for each ERI scale were all over 0.90, revealing a satisfactory convergence between the empirical structure of the data and the theoretical model (details not shown).

**Table 3.** Item-total correlations and Cronbach’s alpha coefficients of the scales of the 19-item ERI questionnaire

Items	Item-total correlations	Alpha if item is deleted	Alpha of scale
Effort			0.75
lot of school work	0.52	0.70	
have to learn too much	0.63	0.66	
schoolwork tiring	0.58	0.68	
pressure of competition for exams	0.45	0.73	
parents’ high expectations	0.39	0.75	
Reward			0.78
slow in finishing schoolwork	0.34	0.78	
do very well at classwork	0.37	0.78	
trouble figuring out the answers	0.42	0.77	
not recognized by teachers	0.54	0.76	
less positive feedback from teachers	0.54	0.76	
satisfied with teachers’ appraisal	0.43	0.77	
help from teachers	0.48	0.76	
unfair treatment from teachers	0.53	0.76	
schoolmates kind and helpful	0.35	0.78	
unfair treatment from schoolmates	0.41	0.77	
not resulting in a good future	0.41	0.77	
Overcommitment			0.62
think about studying	0.43	0.53	
studying still on mind	0.56	0.34	
trouble sleeping at night	0.33	0.67	

**Table 4.** Factor structure of the 19-item Effort–Reward Imbalance questionnaire using principal axis extraction and varimax rotation

Scales	Items	F1	F2	F3	F4
Effort	lot of school work		0.62		
	have to learn too much		0.71		
	schoolwork tiring		0.65		
	pressure of competition for exams		0.47		
	parents’ high expectations		0.42		
Reward	slow in finishing schoolwork			0.59	
	do very well at classwork			0.51	
	trouble figuring out the answers			0.60	
	not recognized by teachers	0.58			
	less positive feedback from teachers	0.60			
	satisfied with teachers’ appraisal	0.50			
	help from teachers	0.58			
	unfair treatment from teachers	0.62			
	schoolmates kind and helpful	0.46			
	unfair treatment from schoolmates	0.49			
	not resulting in a good future	0.39			
Overcommitment	think about studying				0.54
	studying still on mind				0.65
	trouble sleeping at night				0.50
Variance explained (%)		13.09	10.78	6.54	5.99

Only items with factor loading >0.30 are shown.

Table 5 shows how a lack of reciprocity between effort expended at school and subsequent rewards received might be related to a decline in self-rated health. In the first, unadjusted, model (model I) all 3 scales were significantly associated with lower self-rated health, as expected. Combining the 2 core variables into a ratio (upper tertile versus lower tertile) resulted in odds ratios that were double to triple those of the respective scales. Adjusting for sociodemographic and health-related behaviors (models II and III) did not substantially change the odds ratios. However, if family wealth was also taken into account (model IV), the effects were slightly reduced, although still statistically significant (except for the overcommitment scale, for which the association was marginal). In further sex- and grade-specific analyses, the health effects of psychosocial stress at school were stronger in girls than in boys, and stronger in high school students than in middle school students.

**Table 5.** Odd ratios (95% CI) of diminished self-rated health by effort–reward imbalance at school, by sex and grade level.

	Model I	Model II	Model III	Model IV
Effort (per SD)	1.28 (1.10, 1.48)^b^	1.27 (1.09, 1.49)^b^	1.26 (1.08, 1.48)^b^	1.23 (1.05, 1.44)^a^
Reward (per SD)	0.61 (0.52, 0.71)^c^	0.60 (0.51, 0.71)^c^	0.61 (0.52, 0.72)^c^	0.65 (0.55, 0.76)^c^
Overcommitment (per SD)	1.16 (1.01, 1.32)^a^	1.16 (1.01, 1.33)^a^	1.15 (1.00, 1.32)^a^	1.12 (0.98, 1.29)
Effort–reward ratio				
Lower tertile	1.00	1.00	1.00	1.00
Intermediate tertile	1.88 (1.35, 2.63)^c^	1.80 (1.27, 2.56)^c^	1.72 (1.21, 2.45)^c^	1.67 (1.17, 2.39)^c^
Upper tertile	3.52 (2.53, 4.89)^c^	3.39 (2.37, 4.83)^c^	3.25 (2.27, 4.65)^c^	2.80 (1.94, 4.03)^c^
Log (effort/reward) (per SD)	1.86 (1.60, 2.17)^c^	1.85 (1.57, 2.18)^c^	1.81 (1.53, 2.14)^c^	1.67 (1.41, 1.98)^c^

	Sex (fully adjusted model)		Grade (fully adjusted model)	
		
	Boys	Girls	Middle schools (Grade 7–9)	High schools (Grade 10–12)

Effort (per SD)	0.96 (0.76, 1.23)	1.52 (1.22, 1.88)^c^	1.14 (0.89, 1.46)	1.26 (1.04, 1.52)^a^
Reward (per SD)	0.57 (0.44, 0.74)^c^	0.71 (0.57, 0.89)^b^	0.68 (0.54, 0.85)^b^	0.67 (0.55, 0.82)^c^
Overcommitment (per SD)	0.94 (0.76, 1.18)	1.28 (1.06, 1.55)^b^	1.12 (0.90, 1.40)	1.13 (0.95, 1.36)
Effort–reward ratio				
Lower tertile	1.00	1.00	1.00	1.00
Intermediate tertile	1.35 (0.76, 2.38)	2.09 (1.29, 3.30)^c^	1.34 (0.78, 2.30)	1.57 (1.00, 2.47)^a^
Upper tertile	1.98 (1.11, 3.53)^a^	3.16 (1.97, 5.10)^c^	2.54 (1.48, 4.37)^c^	3.15 (2.01, 4.94)^c^
Log (effort/reward) (per SD)	1.41 (1.10, 1.81)^b^	1.95 (1.54, 2.45)^c^	1.60 (1.25, 2.06)^c^	1.67 (1.29, 2.11)^c^

^a^*P* < 0.05, ^b^*P* < 0.01, ^c^*P* < 0.001.

Model I: non-adjusted.

Model II: adjustment for sex, age, and grade level.

Model III: Model II + additional adjustment for smoking, alcohol drinking, and physical activity.

Model IV: Model III + additional adjustment for family wealth.

## DISCUSSION

Our findings show that the effort–reward imbalance model, which was designed to identify a health-adverse psychosocial work environment, can be successfully adapted to school settings. Using a 19-item questionnaire, we replicated the 3 components of the model, and confirmatory factor analysis documented satisfactory fit of the data structure with the theoretical concepts of this cross-sectional study. Importantly, scoring high on the effort-reward ratio, ie, perceiving a marked lack of reciprocity between effort expended and rewards obtained, conferred a 2- to 3-fold risk of diminished self-rated health in this sample of Chinese adolescents. Moreover, we observed sex and grade differences, which is in accord with other research findings. Students in lower grade levels in Europe, North America, and China had much more positive perceptions of their psychosocial school environment (such as school-related stress, schoolmate support, and perceived academic achievement) than those in higher grade levels. Differences between sexes were inconsistent.^9^^,^^13^^,^^17^ It is perhaps unsurprising that Chinese students receiving a high school education—which is no longer compulsory—report worse psychosocial school conditions (including the need for greater effort with lower rewards) than do those in middle school, which remains compulsory, and that a higher health burden might result from these exposures. Nevertheless, given similar levels of exposure to stress in boys and girls, it appears that girls are more vulnerable than boys to the adverse health effects of such stress.^27^

To our knowledge, this is the first report to measure an adverse psychosocial school environment in terms of a theoretical sociological model, ie, the effort–reward imbalance. In addition to possessing satisfactory psychometric properties, these measures were consistently associated with an elevated risk of diminished self-rated health in this large Chinese sample of generally healthy adolescent students. In our analyses, we adjusted for a number of relevant variables, including adverse health behaviors, but the observed effects remained statistically significant. It has been clearly shown that behavioral changes are an indirect pathway from stress to ill health and disease.^28^ In addition, our supplementary analyses reveal significant associations of smoking, alcohol consumption, and physical inactivity with effort–reward imbalance, particularly with the reward component.

In comparison to their peers in other countries, Chinese students are among the most engaged populations in terms of time committed to studying. Our data indicate that 31% of students spent more than 3 hours doing homework on weekdays and 59% did so during weekends, whereas the average percentage of European students who did so was only 19% for weekdays and weekends.^16^ Another indicator of time commitment is daily hours spent watching TV. Our data showed that only 3% of Chinese students reported watching more than 4 hours of TV on weekdays, with 17% doing so on weekends; the figures for European students were 26% on weekdays and 45% at weekends.^16^ Obviously, Chinese adolescents are heavily occupied by school-related work; therefore, they may have little time to commit to issues and concerns outside school. This assumption is supported by the observation that a majority of Chinese student (71%) are physically inactive. In addition, there was a significant correlation between hours spent on homework and the effort–reward ratio (*r* = 0.24, *P* < 0.0001), suggesting that hours spent on homework is a proxy measure of psychosocial stress at school.

Some studies conducted in China indicated that an adverse psychosocial school environment had a negative impact on student health and led to health problems, including psychosomatic symptoms, mental disorders, increased blood pressure, and even suicidal behavior.^9^^–^^13^ Our results should be interpreted in the context of an earlier report showing that a demanding school which offered little control and confronted students with classmate relational problems was associated with an elevated risk of somatic problems and feelings of being stressed.^2^ However, our research adds a relevant new construct, namely, the perceived fairness of exchange between student effort expended at school and the subsequent reward received in the form of academic performance, esteem, and enhanced educational prospects. In accordance with the theoretical model, lack of reward explained a substantial part of variance of health, although overcommitment had only limited explanatory power in our analyses, perhaps because it was an unsatisfactory measure, as reflected by the relatively low Cronbach's alpha coefficient for overcommitment (0.62, 3 items only). The demand–control model was also tested in Chinese adolescents as a measure of school-related stress, but the core concept of the model, control, may not have been culturally appropriate. An examination of the items comprising the control measurement from the WHO HBSC Study (eg, “In our school the students take part in making rules”) shows that the items do not reflect the current sociocultural context of Asian countries such as China. In other words, Asian students typically have little control at school.^29^

According to one recent report, the main sources of school-related stress for Chinese students are heavy schoolwork and homework, teacher pressure, peer pressure, parent pressure, and social norms.^14^ Therefore, the unpleasant reciprocity between student effort expended (schoolwork and expectations) and rewards obtained (academic performance, esteem from teachers and schoolmates, and educational prospects) seems to encompass the essential aspects of an adverse psychosocial school environment, at least in the Chinese educational setting. Although this perceived imbalance does not take into account the level of students’ objective performance, it is nevertheless an important subjective evaluation of inequity that has consequences for individual health and well-being. In the context of paid work, this inequity was shown to predict elevated risks of incident stress-related diseases such as coronary heart disease and depression.^30^^,^^31^ Effort–reward imbalance at work was also associated with elevated psychosomatic complaints and reduced physical and mental functioning in young physicians. One of these studies was carried out in Chinese hospitals.^27^^,^^32^ Similar extensions of this theoretical notion of unfair exchange in core social roles were tested with regard to informal social roles, such as volunteering and providing informal help,^33^ and with regard to patterns of exchange in close social relationships.^34^ In all these instances, reduced health was observed in situations with perceived inequity.^34^^,^^35^

The current report suffers from several limitations. First, as the study design is cross-sectional, we cannot draw any conclusions about causality in reported associations. It may be that students with poor health were often those who performed poorly at school, and they tended to overstate their frustration regarding rewards. Yet, studies have shown that reverse causation is unlikely to account for findings in prospective study designs,^4^ and that additional adjustments for psychological characteristics such as negative affectivity or personality traits did not invalidate the reported associations.^36^ Second, the stability of the effort–reward imbalance questionnaire in school settings was not tested in this study, as it was in previous research on ERI at work.^37^ Third, despite the large sample included in this study, the results are restricted to selected schools of a large city in one country, China. It is not known whether they can be generalized to school settings in other countries. A large international study of school children, the WHO HBSC Study, documented significant variation in core conditions at schools across countries.^16^^,^^17^ Thus, further applications of this approach in other countries are needed, in addition to prospective studies.

Despite these limitations, we were able to confirm satisfactory psychometric properties in using the effort–reward imbalance model to measure psychosocial school characteristics, which have been consistently associated with diminished self-rated health in Chinese adolescents. Based on this well-tested theoretical model, our findings provide specific options to address sources of stress in school settings and to promote a healthy psychosocial environment.

## APPENDIX

The Effort–Reward Imbalance questionnaire measuring psychosocial school environment consisted of the following 19 items. For each question, the subjects chose one from Five-point Likert response categories: strongly disagree; disagree; neither disagree nor agree; agree; strongly agree.

### Effort (5 items)

(1) I have too much schoolwork†. (2) I find that I have to learn too much everyday. (3) I find school work tiring†. (4) I am under pressure because of competition for entrance exams to be promoted to the next grade. (5) My parents expect too much of me at school.

### Reward (11 items)

(1) I am pretty slow in finishing my schoolwork*,†. (2) I do very well at my classwork†. (3) I have trouble figuring out the answers in school*,†. (4) I put a lot of effort into my school work, but I don’t get the recognition I deserve from my teacher(s)*. (5) Even though I am studying harder than some of my schoolmates, I get less positive feedback from my teacher(s)*. (6) I am always satisfied with the teachers’ appraisals of my contributions. (7) When I need help, I can get it from my teacher(s)†. (8) I have been treated unfairly by my teacher(s)*. (9) Most of my schoolmates are kind and helpful†. (10) I have been treated unfairly by my schoolmates*. (11) I do not think that studying hard will improve my future/I think studying is useless*.

### Overcommitment (3 items)

(1) As soon as I get up in the morning, I start thinking about my study problems. (2) Studying rarely leaves my mind; it is still on my mind when I go to bed. (3) If I postpone something that I was supposed to do today, I’ll have trouble sleeping tonight.

* reversed items.

† items derived from the optional package for school settings from the study protocol of the international WHO-HBSC2001/2002 survey.
